# 34. Stemming the Rise in Antibiotic Prescription for Community Acquired Respiratory Infections (ARI) During COVID-19 Pandemic in Singapore General Hospital (SGH)

**DOI:** 10.1093/ofid/ofab466.236

**Published:** 2021-12-04

**Authors:** Shena Yun Chun Lim, Peijun Yvonne Zhou, Daphne Yah Chieh Yii, Kai Chee Hung, Lai Wei Lee, Yi Xin Liew, Jia Le Lim, Li Wen Loo, Narendran Koomanan, Nathalie Grace Sy Chua, Benjamin Pei Zhi Cherng, Siew Yee Thien, Winnie Lee, Lay Hoon Andrea Kwa, Shimin Jasmine Chung

**Affiliations:** Singapore General Hospital, Singapore, Not Applicable, Singapore

## Abstract

**Background:**

In early months of COVID-19 pandemic, SGH recorded a year-on-year increase in antibiotic (ABx) use for community acquired acute respiratory infection (CA ARI) from Feb-Apr 2019 (48.7 defined daily doses (DDD)/100 bed-days) to 2020 (50.8 DDD/100 bed-days). To address concerns of misuse, the antibiotic stewardship unit (ASU) expanded prospective audit feedback (PAF) to CA ARI patients admitted to ARI wards, with low procalcitonin (PCT). PAF was conducted on day 2-3 of ABx, on weekdays. Doctors received feedback to stop/modify when ABx was deemed inappropriate. Here, we describe the impact of ASU’s adaptive approach to curb rising ABx use in patients admitted for ARI during COVID-19 pandemic.

**Methods:**

A Pre- & Post-intervention study was conducted. All patients started on ABx (ceftriaxone/co-amoxiclav/piptazo/carbapenems/levofloxacin) for CA ARI & PCT < 0.5µg/L were analysed. Those who died ≤48h of admission; admitted to intensive care; required ABx escalation; >1 infective sites; complex lung infection were excluded. Primary objective was to compare the proportion of ABx stopped ≤4 days (time to final infection diagnosis) Pre (22/3-18/4/20) & Post (21/4-13/7/20).

**Results:**

184 (Pre) & 528 (Post) ABx courses were analysed. ASU audited 51 (Pre) & 380 (Post) courses with the rest discontinued/discharged before review. Patients were largely similar in both periods; a third had low likelihood of bacterial infection (C reactive protein < 30mg/L). In Post, 73 feedback was given to stop ABx (often because symptoms suggested viral/fluid overload) & 18 to switch to oral ABx. 82 (90%) feedback was accepted. No ABx was restarted ≤48h or deaths ≤30 days due to ARI. 1 patient had *C. difficile* diarrhoea a day after ABx cessation as per ASU feedback.

Proportion of all ABx stopped ≤4 days was higher in Post than Pre [27/184 (15%) vs 152/528 (29%), p< 0.01]. Median duration of therapy of IV ABx was reduced (6.5 vs 3 days, p< 0.01), with corresponding shorter median length of stay (10.5 vs 6 days, p< 0.01).

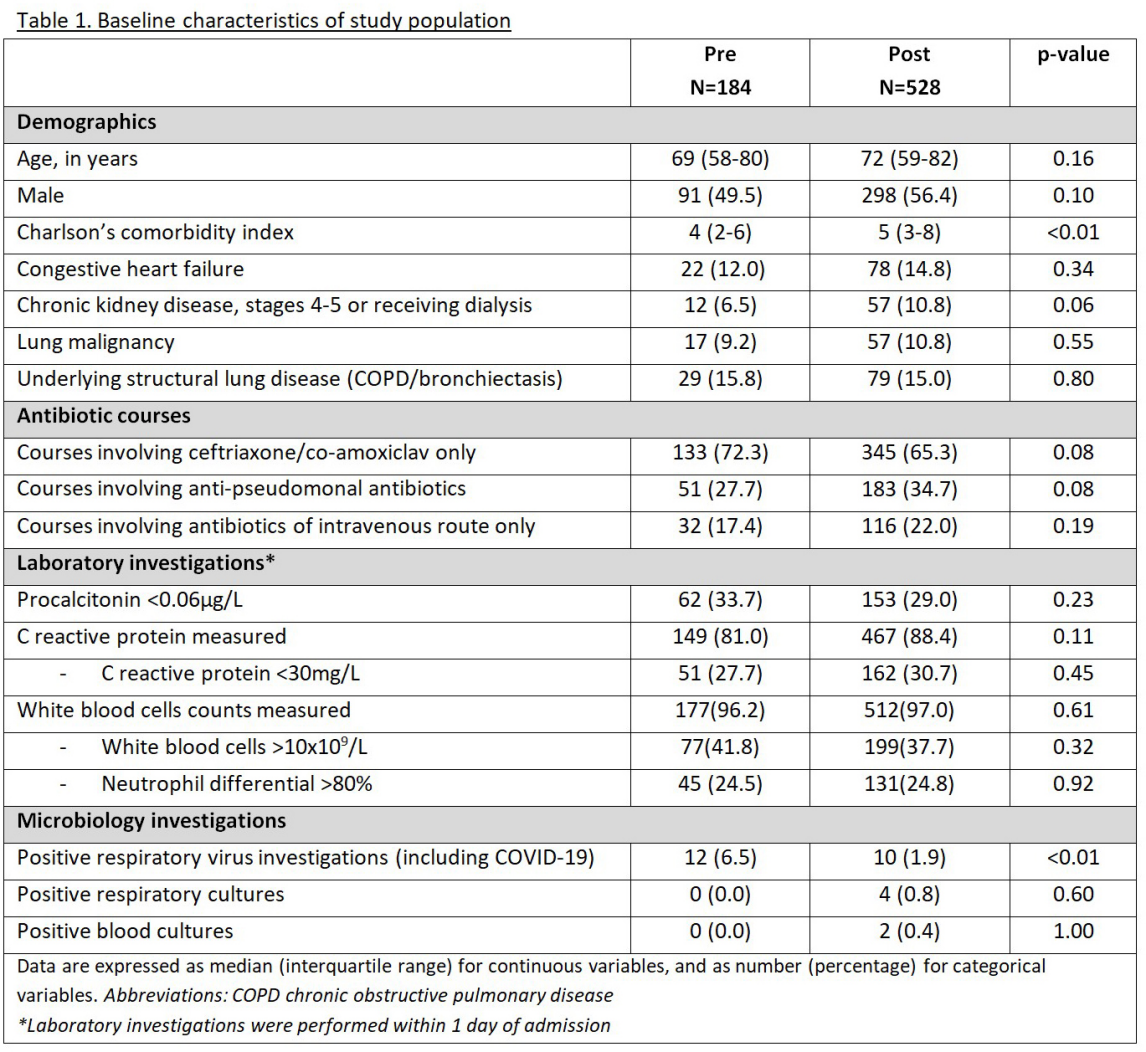

**Conclusion:**

PAF directly and indirectly reduced ABx duration in patients treated for CA ARI as prescribers become more conscious about stopping ABx when investigations show low likelihood of bacterial infection. ASU must remain agile during pandemics to detect emerging problems and adapt processes to counter early.

**Disclosures:**

**All Authors**: No reported disclosures

